# Insensibility Produced by Ethereal Inhalation

**Published:** 1847-03

**Authors:** 


### ARTICLE III.

### *Insensibility produced by Ethereal Inhalation.*

From the Boston Medical and Surgical Journal.

SIR:—Will Mr. Morton's ethereal vapor allay the mental vexation and moral suffering which the papers of his medical supporters have inflicted upon innocent parties, your subscribers, who have been unrighteously cajoled into reading advertisements very like empirical, where they least expected to find them? If this most ancient novelty have such assuaging power, the agent who is now among us to dispense the patent nostrum may do some business. Perhaps even the "ten per cent." might be given for restored equanimity. Good Mr. Editor, how could you let us innocent men be so aggrieved. You know that we are almost driven mad by "patentees." Like Pharaoh's frogs, they are in every man's kneading trough. Through every newspaper—political, religious, literary or miscellaneous—we are assailed by "patentees." In our down-sitting and our uprising, in our houses and in the streets, by printed papers, by flaunting signs, by illuminated letters, by frightful hieroglyphics and by oral applications, we are attacked, out-flanked, surrounded by "patentees;" and now, when driven from all other places of literary entertainment, our jaded minds turn to our private and peculiar journals, lo! even there we are charged to our teeth by "patentees." Truth finds no rest for her foot; the Goths are in the capital; alas! for medicine!

Excuse me, Doctor, if my style is more passionate than precise. This ethereal vapor is in my head, and though, as your recent pages testify, it makes some of your correspondents dull and heavy, steeping their sense in forgetfulness of things once familiarly known, and blinding them to the perception of things obvious to the plainest understanding, it stimulates other minds to a pitch of exaltation a little too high for grave and sententious utterances. But I am somewhat calmer now, and I will endeavor to put my objections to this business in a shape more dialectical than the exordium.

In the first place, I utterly protest against Mr. Morton, or anybody else, "patenting" sulphuric ether—or its application. The law of patents is plain and congruous to common sense. A man may patent an invention. If he makes anything new, in the shape of a machine, or if he forms a new substance, he may patent it. But he cannot apply a known substance to a new use and "patent" the right to use it in this way. For example—we all can make gun cotton. The process is known, it is yours and mine—this gun cotton, with all its powers, is ours, and we may do what we can with it. Well, it has never yet been applied to blasting rocks. I apply it—it succeeds—it proves better than gunpowder. I cannot patent it—I have invented nothing. I have only applied a known power. But suppose I apply it through a particular machine, which enables me to apply the power to advantage. Why, then I may patent my machine. I may claim exclusive right to the means I employ to bring out and make effective the powers of the gun cotton. Prof. Morse cannot patent electro-magnetism, nor can he patent its application to the transmission of intelligence; but he can patent his machine for so using electricity. Suppose I give calomel in a case for which it never was given before, and succeed. Think you, Dr. Smith, that I could forbid you or anybody else to use the drug for a similar purpose? Certainly not. Well, then, suppose I use ether—is the case different? But I need not pursue the argument. The theory of patents is plain, the law is plain, and the case in question is plain.

Secondly, I protest against the whole business, because I verily believe the great discovery to be utterly useless. If it does not succeed better in Boston than in other places nearer to your humble servant, I would not give a farthing for it. In this part of the world it has utterly failed to do what it ought to do, and unfortunately it has done what it ought not to have done. In one instance it produced distressing sensations about the chest, which warned the inhaler to let it alone, and he has a teasing cough placed to the debtor side of the account. In another the patient did not get insensible, but got drunk, and boxed the surgeon-dentist's jaws, as his reward for administering the intoxicating vapor.

But you will say—"the cases! the cases! Dr. Bigelow's cases!

Dr. Warren's cases! the Massachusetts Hospital cases! Are they not satisfactory?" No, Dr. Smith, they are not. They savor prodigiously of "mesmeric" incantation. Do you remember one Dr. Collier, whom a number of the Boston faculty sent to us some years ago, armed with professional certificates, all testifying to his "charming" powers? Pardon me, we are a little suspicious of our Boston brethren since that time. They are clever, men, very clever, but—some of them—a little credulous. Just look at these cases a little closely. "A boy of 16" inhales. "After eight minutes his head falls back." Of course he was insensible, and the dentist proceeded to extract the tooth. But the boy was too wide awake, and made "some resistance to opening the mouth." But he inhaled more, and then the tooth was got out. "At the moment of extraction the features assumed an expression of pain and the hand was raised." Very natural, but not ethereal. When he came too, however, he said he had suffered no pain—"not the slightest consciousness of pain," "had dreamed of Napoleon." From all which it is proved, that people may have pain (evidenced by expression of the features, and movement of the hand) and not feel it.

"A stout boy" inhaled for three minutes—the muscles were relaxed. "During the attempt to force open the mouth" (the muscles being much relaxed) he became conscious—inhaled more, became unconscious—two teeth were extracted. The patient seemed "somewhat" conscious—but on waking he declared it was "the best fun he ever saw," and begged to have another tooth drawn. He was gratified, but this time there was no fun in it, for "the pain" proved to be considerable.

So much for the "stout boy." "A girl of 16" flinched and frowned, but when awake, said she had been dreaming a pleasant dream.

The next patient, "a healthy, middle-aged woman," manifested no pain at all. And these cases, we are told, are "fair examples of the average results produced by the inhalation of the vapor." It is likely they are, and they convince us that ether has precisely the power it was known to have had at least, as we have evidence at hand to show, twenty-five years ago; when in certain cases it produced a degree of narcotism, at the expense of great bronchial irritation, and even, as was then supposed fatal pulmonary disease. Heretofore surgeons have declined using this agent, because they were aware that lethargy and fatal coma were conditions divided by a line of separation too indistinct to permit of the production of insensibility with impunity, and because pulmonary irritation is a more serious evil than pain.

The truth is, that what your correspondents are pleased to call, in medical parlance, "producing insensibility," is, in fact, making people "dead drunk." Every body knows that when a man is in this curious condition he is very insensible to pain. A poor Irishman in this neighborhood, while in this state of "insensibility," had both his legs taken off by a locomotive. He manifested so little "sensibility" that he was not aroused until a surgeon was amputating the shattered stumps, when he cried out "don't be cutting me, me flesh is aisy to hale." Can ethereal vapor beat this exploit of whiskey? I trow not. Now, doctor, if we are to induce insensibility by this class of means, I very much prefer whiskey punch to ether, because it is more certain and more permanent in its effects; it is less dangerous, and, lastly, it will be easier to persuade patients to take it. Moreover, I have the same right to patent whiskey punch for this purpose, that Mr. Morton has to patent ether—for I do not know that such an application of the article has been made before.

But admitting that sul. ether is an invention; admitting that it has all the powers ascribed to it, should the medical and dental professions sanction the patenting of it?

It is mortifying to have to ask this question. I thought it had been settled long since, as a fundamental principle in medical ethics. What do we, any of us, know that we have not received? And shall the debtors to all the great spirits of our time-honored profession refuse to contribute our little mite to the great common treasury of knowledge? Away with such miserable policy as this! Freely we have received, let us freely give. I have not patience to dwell upon this subject. The "cursed thirst for gold" is always detestable, but rarely more so than when it invades a benevolent profession and would speculate in human misery.

Dr. Bigelow's reasons for "patenting" in this case are curious.

Reason I. Because the ether is capable of abuse, and, therefore, "should be restricted to responsible persons." That is, to such as can pay for it.

Corollary 1. People who have money cannot abuse sul. ether.

Corollary 2. Poor people cannot be trusted with knowledge.

Reason II. Its action (that of ether) is not thoroughly understood, "and its use should be restricted to responsible persons" (people who can purchase the right to use it.)

Corollary 1. People who have money can thoroughly understand what is not "thoroughly understood."

Corollary 2. Poor people have poor intelligence.

Reason III. "One of its greatest fields is the mechanical art of dentistry, many of whose processes, are by convention, secret, or protected by patent rights. It is especially with reference to this art that the patent has been secured."

"Producing stupor" belongs to mechanical dentistry. Certainly; who can doubt it. It is lawful to "patent" any thing connected with "mechanical dentistry"—certainly. The profession think otherwise, but no matter: mechanical dentistry differs from other branches of "mechanical surgery," chiefly in this, that it is a fair field for patents. The reason is obvious—to men whose heads are full of ethereal gas.

Corollary. It is lawful to keep secrets from dentists, providing they be imparted to the surgeons of the Massachusetts General Hospital.

Dear Editor, thine ever, T. E. B.

*Baltimore College of Dental Surgery*, *Dec.* 15.

